# Analysis of Extrahepatic Multiple Primary Malignancies in Patients with Hepatocellular Carcinoma according to Viral Infection Status

**DOI:** 10.1155/2012/495950

**Published:** 2012-12-01

**Authors:** Keita Kai, Atsushi Miyoshi, Kenji Kitahara, Masanori Masuda, Yukari Takase, Kohji Miyazaki, Hirokazu Noshiro, Osamu Tokunaga

**Affiliations:** ^1^Departments of Pathology and Microbiology, Faculty of Medicine, Saga University, Nabesima 5-1-1, Saga City 849-8501, Saga, Japan; ^2^Department of Surgery, Faculty of Medicine, Saga University, Saga City 849-8501, Saga, Japan

## Abstract

Previous studies have investigated extrahepatic multiple primary malignancy (EHPM) associated with hepatocellular carcinoma (HCC). However, its correlation with viral infection, such as hepatitis B virus (HBV) or hepatitis C virus (HCV), has not been examined. The aim of this study is to investigate the association between EHPM and hepatitis infection in HCC patients. A total of 412 patients who underwent surgical resection for primary HCC were enrolled. Viral infection was evaluated by serum HBV surface antigen (HBs Ag) and HCV antibody (HCV Ab). Sixty-eight (16.5%) patients had one or more EHPM. The most frequent EHPM was gastric cancer (*n* = 32) in this cohort. No statistical significance was observed in the distribution of viral infection and incidence of entire EHPM. However, HCV Ab, HBs Ag, and negative status for both were correlated with the frequency of gastric (*P* = 0.0194), urinary tract (*P* = 0.0067), and breast cancer (*P* = 0.0036), respectively. Infection of *Helicobacter pylori* was investigated by immunohistochemistry in gastric EHPM and resulted that 20 out of 21 analyzed cases were negative for *Helicobacter pylori*. Although it should be verified by well-designed large cohort studies, the current results suggested correlation between HCV infection and gastric cancer, HBV infection and urinary tract cancer and viral hepatitis-free status and breast cancer in HCC patients.

## 1. Introduction

Extrahepatic multiple primary malignancy (EHPM) associated with hepatocellular carcinoma (HCC) has been investigated in previous reports [1–15]. However, the majority of these studies were performed decades ago. The improved survival rates in HCC patients, advances in diagnostic modalities and clinical treatments, and long-term followup might produce a higher incidence of EHPM as well as useful information for the treatment of HCC patients. Although chronic infection with hepatitis B virus (HBV) and/or hepatitis C virus (HCV) are well-known risk factors and are the most influential determinants for HCC, there have been few investigations of the correlation between viral infection status and EHPM. The aim of this study is to reveal the characteristics of EHPM in a cohort receiving long-term followup as well as the association between EHPM and the viral infection status in HCC patients.

## 2. Materials and Methods

### 2.1. Patients

 A total of 412 patients, who underwent a surgical resection for primary HCC between 1984 and December 2010 at Saga University Hospital, were included in this study. Informed consent for the use of medical information was obtained from all patients and the study protocol was approved by the Ethics Committee of the Faculty of Medicine at Saga University. The HCC histology and the status of liver cirrhosis were pathologically confirmed. No patient who had received liver transplantation was included. The diagnosis of EHPM was based on the criteria of Warren and Gates [16]: (i) each tumor must present a definitive malignancy; (ii) each must be distinct; (iii) the probability of one being a metastasis of the other must be reasonably excluded. Multiple cancers occurring in the same organ were identified as a single cancer in that organ. EHPM cases were classified as “synchronous” if diagnosed within 1 year before or after hepatectomy. EHPM cases occurring more than 1 year before or after hepatectomy were classified as “metachronous”. The viral infection status was evaluated by serum HBV surface antigen (HBs Ag) and HCV antibody (HCV Ab).

### 2.2. Immunohistochemistry of the *Helicobacter Pylori*

*Helicobacter pylori* infection status in the patient with gastric cancer was investigated by immunohistochemistry. The 4 *μ*m sections of formalin-fixed and paraffin-embedded tissues of nontumorous gastric mucosa which were obtained by gastrectomy or endoscopic biopsy were prepared. The primary antibody used was rabbit polyclonal antibody against *Helicobacter pylori* (Clone B0471; 1 : 30 dilution, Dako Cytomation, Glostrup, Denmark). Envision + System (Dako Cytomation) was used as the second antibody. The slides were visualized by diaminobenzidine tetrahydrochloride and the nuclei were counterstained with Hematoxylin. The staining was performed with positive-control slides. The accuracy of staining and the results were confirmed by three pathologists (MM, YT, and KK).

### 2.3. Statistical Analysis

Statistical analyses were performed using the JMP version 8 software package (SAS Institute, Cary, NC, USA). Statistical analysis was performed using Student's *t*-test, the Pearson's chi-square test, and Fisher's exact test, as appropriate. Survival analyses were performed using the Kaplan-Meier method with overall survival determined from the time of hepatectomy to the time of death or most recent followup. Differences in survival curves were compared using the log-rank test. A value of *P* < 0.05 was considered to be statistically significant.

## 3. Results

### 3.1. Clinicopathological Features of the HCC-Only and EHPM Groups

**Figure 1:**
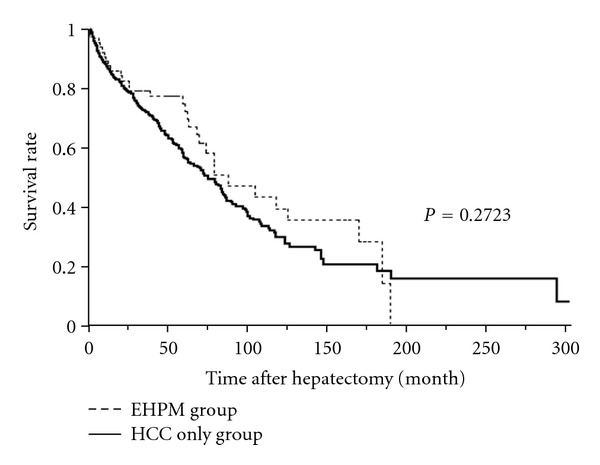
Comparison of Kaplan-Meier curves in HCC-only and EHPM groups. No significant difference was observed in overall survival between the groups (log-rank test, *P* = 0.2723).

Table 1 demonstrates the clinicopathologic features of the HCC-only and EHPM groups. One or more EHPM were found in 68 (16.5%) of 412 patients with HCC. The mean age in the EHPM group was 69.2 ± 8.2 (mean ± standard deviation [SD]) and was significantly higher than that in the HCC-only group, which was 63.9 ± 10.6 (mean ± SD, *P* = 0.0001). The rate of accompanying liver cirrhosis was significantly lower in the EHPM group (31.3%) than the HCC-only group (51.3%; *P* = 0.0025). The sex distribution, alcohol intake, smoking habit, rate of diabetes mellitus, distribution of viral infection, and rate of nonalcoholic steatohepatitis (NASH) were not significantly different in the two groups. Synchronous, metachronous, and multiple EHPMs and viral infection status are shown in Table 2. Cases of coexistent synchronous and metachronous EHPM are categorized as metachronous. The analysis showed that 4.4% (*n* = 18) had synchronous EHPM and 11.9% (*n* = 49) had metachronous EHPM. Four patients had multiple EHPMs. Three of those patients had two organs with EHPMs (gastric + prostate, bladder + leukemia, and esophageal + prostate) and the remaining patient had four organs with EHPMs (gastric + colorectal + bladder + lung). No significant difference was observed between viral infection status and each EHPM category (incidence, synchronous, metachronous, and multiple cases). A comparison of the Kaplan-Meier curves in the HCC-only and EHPM groups are performed (Figure 1). No significant difference was observed in the overall survival between the two groups (log-rank test, *P* = 0.2723).

### 3.2. Site of EHPM and Viral Infection Status

**Figure 2:**
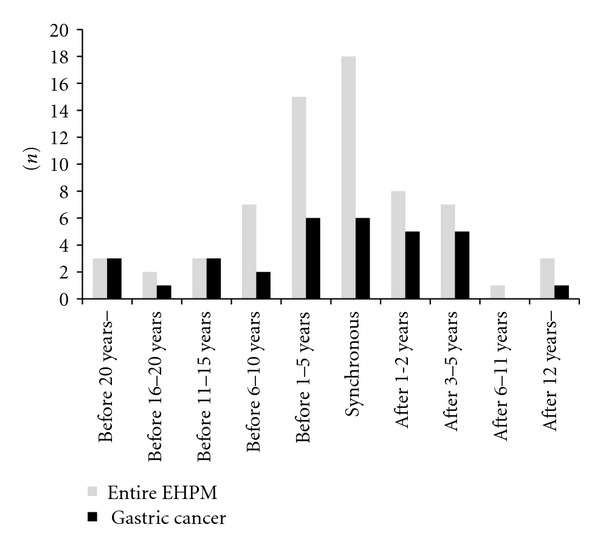
Distribution of EHPM according to time-flow. The EHPMs distributed most in synchronous cases, secondary distributed within before or after 5-years from hepatectomy. Longer the time is away from hepatectomy, more less the EHPM cases have been detected. The same tendencies were observed in urinary tract and breast EHPMs.

 The distribution of organic site and viral infection status among EHPM patient group are shown in Table 3. Each malignancy in multiple EHPM case is separately counted (74 EHPM in 68 patients). The most frequent EHPM in this cohort was gastric cancer, which represented 47.8% (32/74) of all EHPMs. The distribution of viral infection status was significantly different in gastric cancer (*P* = 0.0424), urinary tract cancer (*P* = 0.0002) and breast cancer (*P* = 0.0023) by Pearson's chi-square test. Gastric cancer (*n* = 32, male 28, female 4) was predominantly observed among HCV Ab-positive patients, urinary tract cancer (*n* = 6, all male) was predominant in HBs Ag-positive patients, and breast cancer (*n* = 5, male 1, female 4) was predominant in patients negative for both HCV Ab and HBs Ag. Two of five breast cancer cases met the clinical and pathological criteria for NASH. The analysis was among the four groups and contained small groups with less than five patients limiting relevance of chi-square test. Therefore, further analyses compared between two groups such as gastric cancer and the presence or absence of HCV Ab, urinary tract cancer and the presence or absence of HBs Ag, and breast cancer and both HCV Ab- and HBs Ag-negative or either HCV Ab- or HBs Ag-positive were performed. The results and calculated odds ratios with 95% confidence intervals (CI) are shown in Table 4. Although the sample size of urinary tract cancer patients and breast cancer patients were still small, HCV Ab, HBs Ag, and negative status for both were correlated with the frequency of gastric (*P* = 0.0194), urinary tract (*P* = 0.0067), and breast cancer (*P* = 0.0036), respectively, (by two-sided Fisher's exact test).  Figure 2 shows the distribution of EHPM according to time-flow. EHPMs were distributed most in synchronous cases, secondary distributed within before or after 5-years from hepatectomy. The longer the time since hepatectomy, the fewer the EHPM cases were detected. The same tendencies were observed in gastric, urinary tract, and breast EHPM. One of three cases of gastric EHPM treated more than 20 years ago received a blood transfusion during gastrectomy and subsequently contracted hepatitis C.

### 3.3. Clinicopathologic Features of Gastric EHPM and *Helicobacter Pylori* Infection Status

 Because of the sample size, clinicopathological analysis was performed only for the cases of gastric EHPM. 84.4% (27/32) of the gastric EHPM cases were detected in the early stage of disease. The most common histological type was the intestinal type based on Lauren's classification (21/32, 65.6%). Five cases were the diffuse type in Lauren's classification and the histological types of the remaining six cases were unknown. Of patients with gastric EHPM, 68.8% (22/32) underwent gastrectomy while the remaining 31.2% (10/32) underwent endoscopic resection. No significant difference was observed between HCV Ab-positive patients and HCV Ab-negative patients in terms of stage, histology, and treatment.

 Of the 32 cases of gastric EHPM, 21 cases of gastric tissue were available. Using these specimens, immunohistochemistry of *Helicobacter pylori* was performed. Unexpectedly, 20 cases out of 21 analyzed cases (92.5%) were negative for *Helicobacter pylori*. A small amount of *Helicobacter pylori* was detected in remaining one case.

## 4. Discussion

 The frequency of the EHPM site in HCC patients varies according to the geographical setting of the study. Genitourinary (mainly prostate) and colorectal tumors are described as the most prevalent sites in Western series [1, 5, 9, 11, 13, 15]. Previous Japanese studies consistently reported gastric cancer as the most frequent EHPM in HCC patients [2–4, 10, 14]. In fact, gastric cancer is occasionally detected during clinical followup for HCC, and thus this theme was focused in the present study.

 Chronic infection with HBV and/or HCV is a risk factor and the most influential determinant for HCC. However, the relationship between EHPM and viral hepatitis in patients with HCC has not been thoroughly examined in previous studies. The systemic effects of viral infection may play a role in development of EHPM. Bruno et al. hypothesized that HCV infection could play an important role not only in the development of HCC, but also of EHPM, based on the finding that all five of their EHPM cases were HCV Ab-positive [13]. Several studies have noted HCV infection might be involved in the pathogenesis of B-cell lymphomas [17, 18]. Di Stasi et al. reported that the most common EHPM in their HCC cohort was B-cell lymphoproliferative disorders, with a higher incidence than expected in the reference population, and that 50% of patients with B-cell lymphoproliferative disorders had an HCV infection [9]. However, most other series reported relatively low incidence of B-cell lymphoproliferative disorders in HCC patients [1–5, 7, 8, 10, 11, 15].

The prevalence of hepatitis virus infection also varies geographically; the prevalence of chronic hepatitis B infection is particularly high (10–20%) in China and in sub-Saharan Africa, while it is low (0.2–0.5%) in North America, Northern, Western, and Central Europe, and Australia [19]. HCV also has clear differences in genotype prevalence in different geographic regions [20]. Around 80% of HCC cases in Japan are related to HCV infection (approximately 70% of infections are caused by genotype 1b), but the prevalence of HBV is relatively low [21]. The incidence of gastric cancer in Japan is extremely high in comparison to that in Western countries [22]; therefore, the reported high incidence of gastric cancer among Japanese patients with HCC may simply reflect the high incidence of gastric cancer in general in the Japanese population. However, the current study found that most of these gastric cancers were not associated with *Helicobacter pylori* infection which is considered as one of the main cause of the gastric cancer. A study from Taiwan has also reported gastric cancer as the most common EHPM among HCC patients [7]. The most common HCV genotype in Taiwan is 1b (50% to 70%), which is similar to the situation in Japan [23, 24]. This may suggest a relationship between gastric cancer and HCV, especially for infection with genotype 1b.

 One unexpected finding was that HBV infection showed the tendency of correlation with urinary tract cancer (urothelial carcinoma) in this study. The association between HBV and urothelial carcinoma has not been thoroughly investigated. A few reports presented suggestive evidence for a relationship of HBV infection and urothelial carcinoma. A report from Taiwan documented that the most common postrenal transplantation malignancies are HCC and urothelial carcinoma and that the high rate of HBV and HCV in Taiwan may explain this finding [24]. One case report describes HCC and urothelial carcinoma that synchronously developed in a patient with a chronic hepatitis B infection [25]. Identification of a relationship between HBV infection and the pathogenesis of urothelial carcinoma would be clinically important. Cohort studies from other countries, especially from areas with a high prevalence of chronic hepatitis B infection, such as China and sub-Saharan Africa, would provide important data to help this potential relationship.

 Another unexpected finding was that breast cancer showed the tendency of correlation with a lack of infection with hepatitis B or C. It is interesting that two of five breast cancer cases were identified as having NASH. Although the incidence of breast cancer in nonalcoholic fatty liver disease (NAFLD) including NASH patients has not been clarified, there is a well-established link between obesity and postmenopausal breast cancer. One hypothesis is that this is because of an increase in the serum concentration of bioavailable estradiol [26].

 In conclusion, this study analyzed EHPM status in HCC patients with regard to infection with HBV and/or HCV. The results suggested that gastric cancer, the most common EHPM based on previous Japanese reports, was associated with HCV-infection in this Japanese cohort. In addition, relationships between HBV infection and urothelial carcinoma and a lack of infection with hepatitis virus and breast cancer were also suggested. However, this study involved following limitations. The information about viral activity (HBV-DNA and HCV-RNA) was not available in this cohort and the sample size was too small especially in the urothelial carcinoma and the breast cancer patients. A community-based cohort study would be appropriate for this kind of association study. Therefore, the current results should be verified by well-designed large cohort studies in the future.

## Figures and Tables

**Table 1:** Clinical characteristics of HCC-only and EHPM groups.

	HCC only group (*n* = 344)	EHPM group (*n* = 68)	*P* value
Age (mean ± SD)	63.9 ± 10.6	69.2 ± 8.2	0.0001
Male/Female (ratio)	264/80 (3.3 : 1)	56/12 (4.7 : 1)	0.3434
Viral infection status			0.7641
HCV Ab (+)/HBs Ag (−)	221 (64.2%)	46 (67.7%)	
HCV Ab (−)/HBs Ag (+)	57 (16.6%)	8 (11.8%)	
HCV Ab (+)/HBs Ag (+)	7 (2.0%)	2 (2.9%)	
HCV Ab (−)/HBs Ag (−)	59 (17.2%)	12 (17.7%)	
NASH^†^	8 (2.3%)	3 (4.4%)	0.3295
Alcohol abuse	44 (12.8%)	10 (14.7%)	0.6689
Smoking history	197 (57.3%)	36 (54.4%)	0.6640
Diabetes mellitus	84 (24.4%)	12 (17.7%)	0.2274
Liver cirrhosis^‡^	174 (51.3%)	21 (31.3%)	0.0025

HCC: hepatocellular carcinoma; EHPM: extrahepatic primary malignancy; SD: Standard deviation; N.S.: Not significant; HBs Ag: hepatitis B virus surface antigen; HCV Ab: hepatitis C virus antibody; NASH: nonalcoholic steatohepatitis.

^†^Assessed by clinical data and pathological assessment of surgically resected liver specimens.

^‡^Assessed by surgically resected liver specimens.

**Table 2:** Synchronous, metachronous, and multiple EHPM and viral infection status.

	HCV Ab (+)/HBs Ag (−)	HCV Ab (−)/HBs Ag (+)	HCV Ab (+)/HBs Ag (+)	HCV Ab (−)/HBs Ag (−)	Total
	*n* = 267 (%)	*n* = 65 (%)	*n* = 9 (%)	*n* = 71 (%)
Incidence of EHPM	46 (17.2)	8 (12.3)	2 (22.2)	12 (16.9)	68 (16.5)
Synchronous	8 (3.0)	3 (4.6)	1 (11.1)	6 (8.5)	18 (4.4)
Metachronous^†^	38 (14.2)	5 (7.7)	1 (11.1)	5 (7.0)	49 (11.9)
Multiple EHPM	2 (0.75)	1 (1.5)	0	1 (1.4)	4 (0.97)

^†^Coexisting synchronous and metachronous EHPM is categorized as metachronous.

No statistical significance was observed in distribution by viral infection status of each EHPM category.

**Table 3:** Site of EHPM and viral infection status.

Site of EHPM (histology)	HCV Ab (+)/HBs Ag (−)	HCV Ab (−)/HBs Ag (+)	HCV Ab (+)/HBs Ag (+)	HCV Ab (−)/HBs Ag (−)	Total (patients)^†^	*P *value
	*n* = 50 (%)	*n* = 9 (%)	*n* = 2 (%)	*n* = 13 (%)	*n* = 74 (%)	
Gastric (ad)	27 (54.0)	2 (22.2)	0	3 (23.1)	32 (43.2)	0.0424
Head and neck (scc)^‡^	4 (8.0)	1 (11.1)	0	1 (7.7)	6 (8.1)	0.9538
Urinary tract (uc)^§^	2 (4.0)	4 (44.4)	0	0	6 (8.1)	0.0002
Prostate (ad)	3 (6.0)	0	1 (50.0)	2 (15.4)	6 (8.1)	0.1018
Breast (dc)	1 (2.0)	0	0	4 (30.8)	5 (6.8)	0.0023
Colorectal (ad)	3 (6.0)	1 (11.1)	0	1 (7.7)	5 (6.8)	0.8893
Kidney (rcc)	1 (2.0)	0	1 (50.0)	1 (7.7)	3 (4.1)	0.1502
Extrahepatic biliary tract (ad)^¶^	1 (2.0)	0	0	1 (7.7)	2 (2.7)	0.6548
Skin (scc)	2 (4.0)	0	0	0	2 (2.7)	0.8048
B-cell lymphoma	2 (4.0)	0	0	0	2 (2.7)	0.8048
Uterus (unknown)	2 (4.0)	0	0	0	2 (2.7)	0.8048
Pancreas (dc)	1 (2.0)	0	0	0	1 (1.4)	0.9221
Lung (ad)	1 (2.0)	0	0	0	1 (1.4)	0.9221
Leukemia	0	1 (11.1)	0	0	1 (1.4)	0.0548

^†^Each malignancy in multiple EHPM case is separately counted. ^‡^Four cases of esophagus, one case of larynx and one case of oral. ^§^Five cases of bladder and one case of ureteral. ^¶^One case of gallbladder and one case of bile duct. ad: Adenocarcinoma, scc: Squamous cell carcinoma, uc: Urothelial carcinoma, dc: Ductal carcinoma, rcc: Renal cell carcinoma.

**Table 4:** Odds ratios for infection-associated EHPM^†^ according to viral infection status.

	Infection-associated EHPM (+)	Infection-associated EHPM (−)	Odds ratio (95% CI)	*P* value
HCV Ab (+)	27	240	3.15 (1.19–8.37)	0.0194
HCV Ab (−)	5	140		
HBs Ag (+)	4	61	11.31 (2.03–63.11)	0.0067
HBs Ag (−)	2	345		
No viral infection	4	67	20.30 (2.23–184.46)	0.0036
Any viral infection	1	340		

^†^Gastric cancer for the presence or absence of HCV Ab, urinary tract cancer for the presence or absence of HBs Ag, breast cancer for both HCV Ab- and HBs Ag-negative or either HCV Ab- or HBs Ag-positive.

EHPM: Extrahepatic primary malignancy, HBs Ag: Hepatitis B virus surface antigen, HCV Ab: Hepatitis C virus antibody, CI: Confidence interval.
